# Review: Nicotinic acetylcholine receptors to regulate important brain activity—what occurs at the molecular level?

**DOI:** 10.1007/s11571-023-09975-7

**Published:** 2023-05-19

**Authors:** Shigetoshi Nara, Yutaka Yamaguti, Ichiro Tsuda

**Affiliations:** 1 Graduate School of Natural Science and Technology, Okayama University, 3-1-1 Tsushima-naka, Kita-ku, Okayama, 700-8530 Japan; 2 Faculty of Information Engineering, Fukuoka Institute of Technology, 3-30-1 Wajiro-higashi, Higashi-ku, Fukuoka, 811-0295 Japan; 3 Chubu University Academy of Emerging Sciences/Center for Mathematical Science and Artificial Intelligence, Chubu University, Aichi, 487-8501 Japan

**Keywords:** Neuromodulator, Nichotinic, Acetylcholine, Receptors, Brain activity

## Abstract

Herein, we briefly review the role of nicotinic acetylcholine receptors in regulating important brain activity by controlled release of acetylcholine from subcortical neuron groups, focusing on a microscopic viewpoint and considering the nonlinear dynamics of biological macromolecules associated with neuron activity and how they give rise to advanced brain functions of brain.

## Introduction

**Fig. 1:**
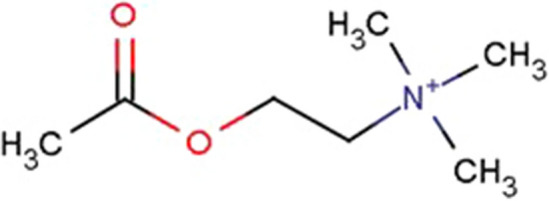
Acetylcholine molecule. The molecular weight is 146

It is well known that neuromodulators play very important roles in advanced functions of brain. Acetylcholine (ACh) is not a highly complex molecule but a rather simple one, as shown in Fig. 1 and it works as a functional neuromodulators. Furthermore, it is related to the various advanced functions of brain, such as perception, learning, memory, and attention, etc. [1–3]

**Fig. 2:**
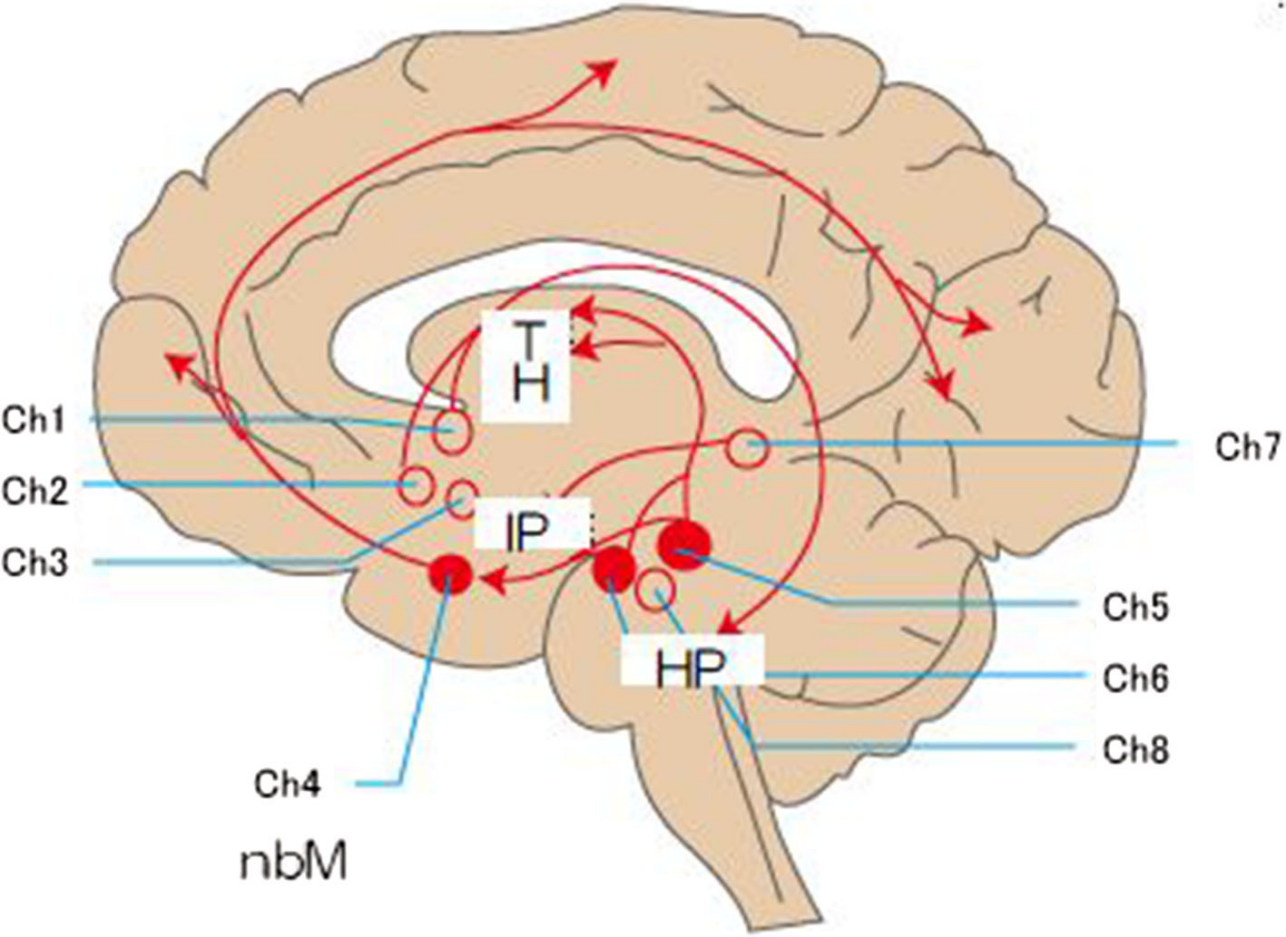
The configuration of the 8 cholnergic cell groups (Ch1 $\sim$ Ch8) in the brain and their projecting axons that release ACh in selected areas in the brain (from https://kanri.nkdesk.com/). TH: thalamus, IP: interpeduncular nucleus, HP: hippocampus. It should be noted that  the atnatomical positions of Hippocampus (HP) and of IP shown in this website should be located a little higher and lower than the described ones, respectively

In the human body, particularly in the brain, acetylcholine is released by neural cells called “cholinergic cells” that exists in subcortical regions. There are 8 cholinergic cell groups in the brain that release ACh; these groups are designated Ch1 $\sim$ Ch8. Regarding visual perception processing, a particularly important cell group is the nucleus basalis of Meynert (nbM) in the basal forebrain; its long projecting axons release controlled amounts of acetylcholine to very wide area of neocortical regions in the frontal, parietal, temporal, and even occipital lobes (see Fig. 2).

The reason why acetylcholine has such strong influence on various advanced functions is that, once ACh is weakly adsorbed at specific sites on cell-membrane-embedded biological macromolecules called “receptors”, it activates various signal transduction pathways inside the cell [4]. The specific receptors that respond to ACh are known as “acetylcholine receptors” (AChRs hereafter). It is known that there is a wide variety of AChRs, as briefly introduced below.

**Fig. 3:**
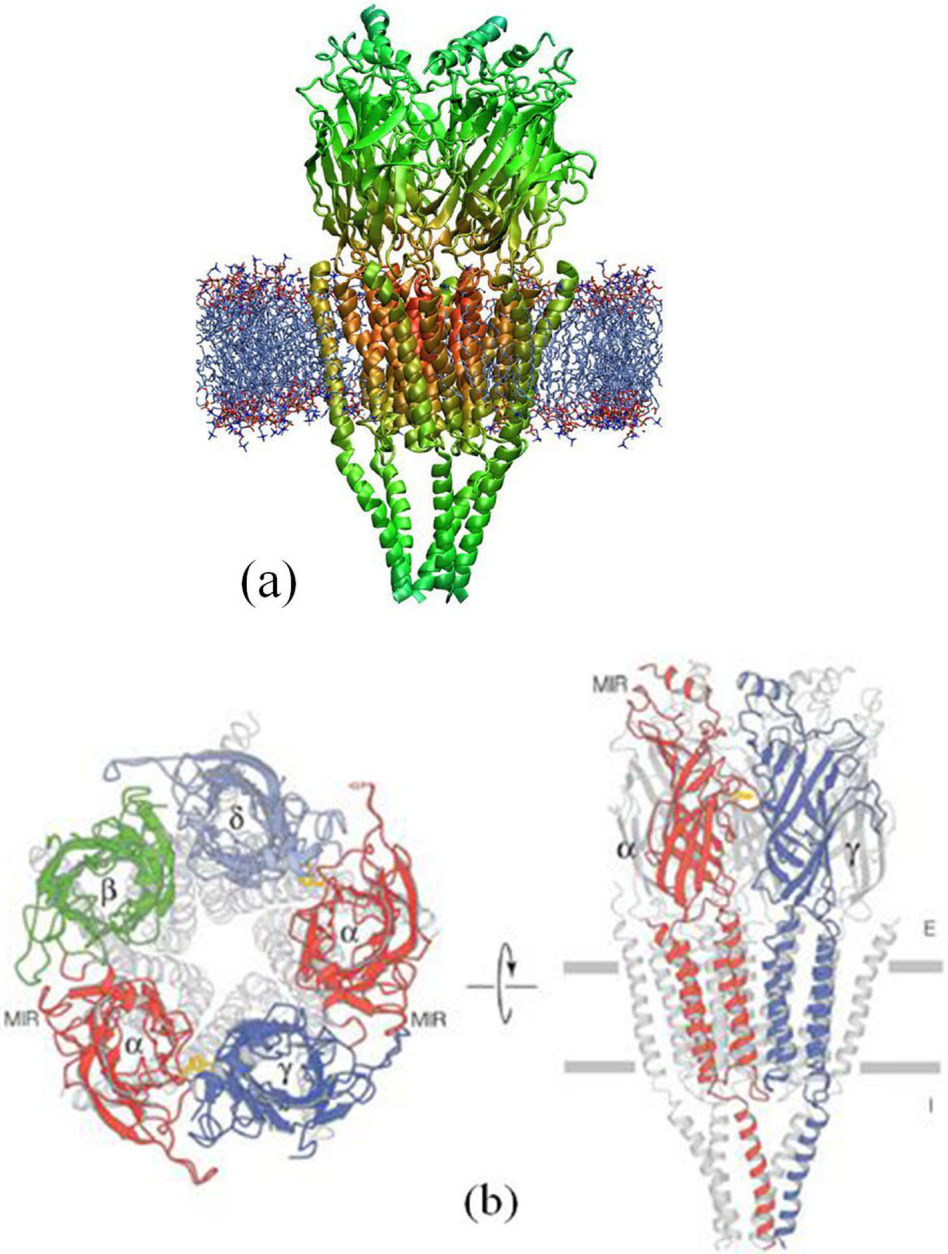
Typical molecular structure of nAChR described in the following papers. **a** [7] **b** [8]. The estimated largest diameter of the cylindrical molecule is approximately $\sim$10nm or more

AChRs are classified into two major types: the nicotinic Acetylcholine Receptor (nAChR) [5] and the muscalinic Acetylcholine Receptor (mAChR) [6]. In the present paper, we will confine ourselves to nAChR as a matter of convenience, given the limited space available. nAChRs are pentamers consisting of five subunits, each of which is a trans-membrane proteins; the total molecular weight of the five subunits is about 250,000 $\sim$ 350,000, and each of the five subunits is a 4-transmembrane-domain protein. Typical experimental observations and their computer reconstructions are shown in Fig. 3 [7–9]. To date, despite a long history of investigation into the pertinent mechanisms of acetylcholine receptors, we do not yet have fully clarified the roles of AChRs in advanced brain functions [1].

## Phenomena at the microscopic level

On cytoplasmic membrane of neural cells, there are many transmembrane proteins to mediate signals transmission. In such proteins, our concern in this paper is nAChRs introduced in Introduction.

**Fig. 4:**
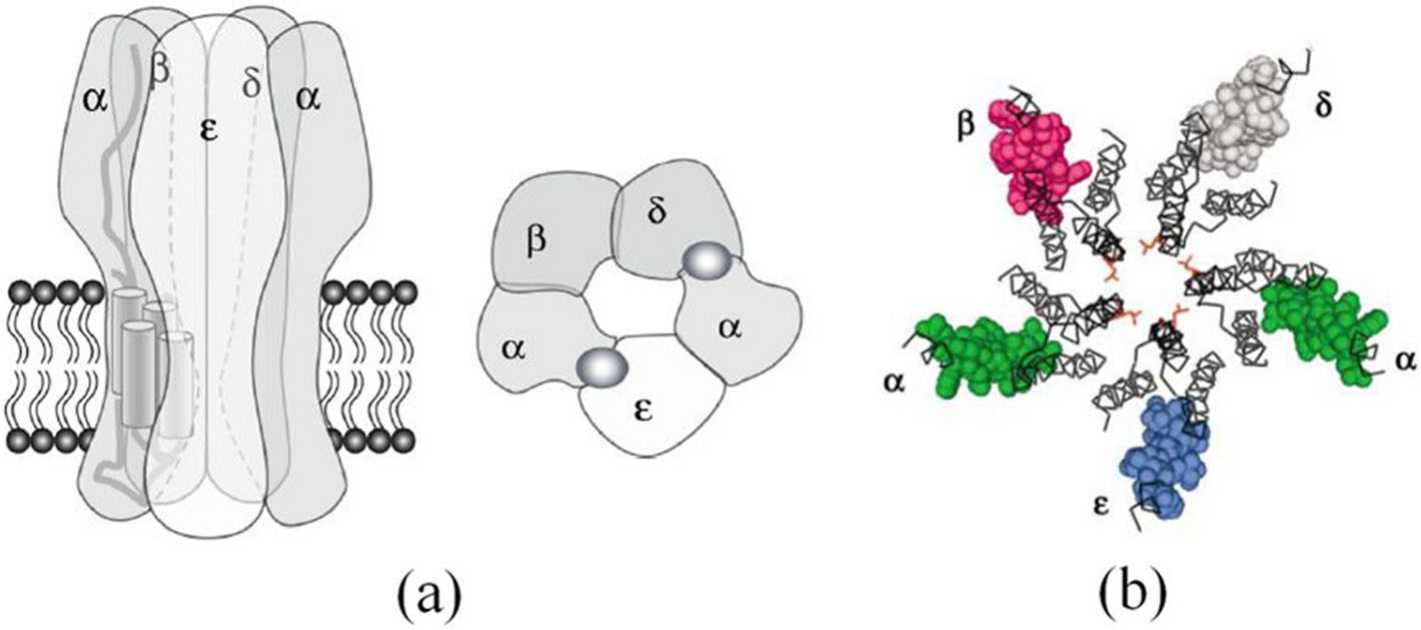
Configuration of subunits and intra subunit structures to form ion-channel as reported by **a** [12], **b** [13]. The two binding sites of ACh are shown in **a** by the dense grey circle. In **b** at the center of pentagon, there are the five M2-$\alpha$-helices to form ion-channel

**Fig. 5:**
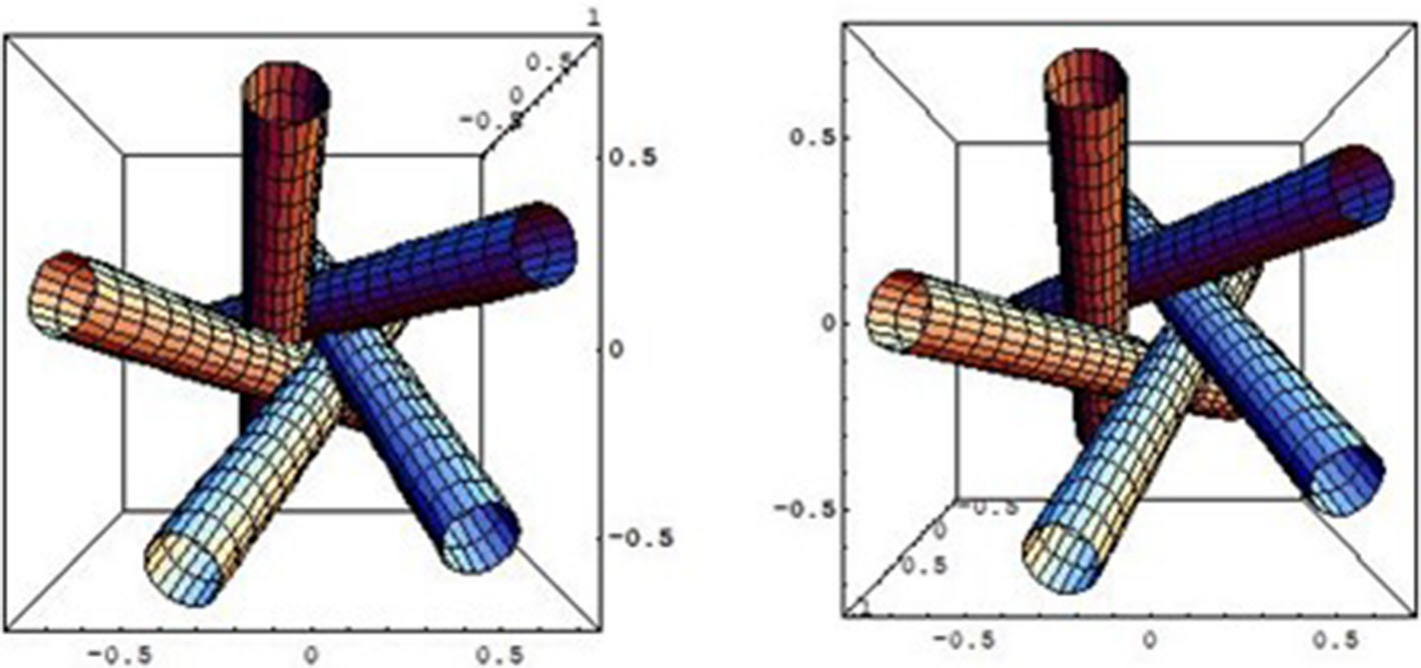
Schematically described pentagonal configuration consisting of M2-$\alpha$-helix in five subunits of nAChR drawn by one of the authors (S.N.) with use of Mathematica. The left is closed state and the right is open state of ion-channel. In the former, the upper pentagon formed by the top of five cylinders and the lower pentagon formed by the bottom of five cylinders are aligned in symmetric configuration. In the latter, the upper pentagon and the lower pentagon are rotated slightly, such that a narrow gap opens

nAChRs belong to a group of proteins called “ion channel proteins”. One of the reasons why nAChRs are of particular interest to the research community, including our team, is that they *regulate neuron firing* by switching their functional state, such as paying attention, memorizing, wakefulness, etc., in a controlled manner to allow sodium ions to pass from the outside to the inside of cell through tiny hollow channel, the diameter of which is on a sub-nanometer scale; the channel has the gate to take either “the open state” or “the closed state”. The most important point is that the both states are controlled by binding or unbinding of acetylcholine to specific sites on receptors (see Figs. 4 & 5) [10–13]. The papers on this topic is too extensive to discuss within the space limitations here. Readers can familiarize themselves with a large body of experimental data and numerous in-depth considerations through the excellent book written by Changeux & Edelstein [14].

## Molecular structure and problems

It is known that nAChRs are pentameric in structure and constitute a quite large family depending on different combinations of various kinds of subunits, which are classified into five types according to their amino-acid-sequences: $\alpha , \beta , \gamma , \delta$, and $\epsilon$. Of these, the first two have intra-subunit variations, i.e. $\alpha 1 \sim \alpha 10$ and $\beta 1 \sim \beta 4$. The others, $\gamma , \delta$, and $\epsilon$, do not have distinct subtypes [15–17]. Depending on the combination of subunits in the pentamer, permeability of ion are different under acetylcholine binding, and they are regarded as important factors to regulate advanced functions of brain [1].

Now, let us summarize the important problems regarding these nAChRs. First, from a microscopic viewpoint and nonlinear dynamics of molecular structure, the following problems exist:

1. The relaxation time problem, i.e. molecular weights of the receptors are distributed over 250,000$\sim$350,000, while the molecular weight of acetylcholine is only 146 and is three or more order of magnitude less than receptors. Thus, binding of acetylcholine to receptors is regarded as a very small physicochemical impact and, in conventional viewpoint of thermal fluctuation based on statistical mechanics, the influence may be relaxed within, at most, nanosecond order. However, after a delay on the order of microseconds following binding, a conformation change in the pentagonal configuration of subunits occurs, and the ion channel at the center of pentagon opens.
2. The spatial coherency problem, i.e., the binding position of acetylcholine is far from the position of ion channel, more than $\sim$10nm, a distance that seems to be too far to keep dynamically coherent signal transmission in conventional viewpoint of statistical physics in equilibrium systems.
3. The energy source problem, i.e., the conformation change associated with ion channel opening is a slight but meaningful distortion and shifting of five M2-$\alpha$-helices belonging to each subunit. Thermal fluctuation energy or physicochemical energy (adsorbing energy) of ACh is highly probably not enough to cause such movement of five helices.

These are major issues not only in biophysics, biochemistry, and molecular biology [18], but also in nonlinear & non-equilibrium dynamics of systems with huge but finite degrees of freedom related with ‘fluctuation theorem’ that has been extensively discussed since 1993 [19].

The second problem is the complex, entangled relation between releasing control of ACh by cholinergic cell groups in the brain, spatial distribution of nAChRs on neural cells in the brain, and concentration control of acetylcholinesterase (degrading enzyme of ACh). These three factors govern the total activity of neural networks in the brain and their complex hierarchical regulatory structures from microscopic scale to macroscopic scale are almost out of consideration to deal with the conventional methods.

Extremely rough estimation with respect to the number of neural cells and synaptic connections in human brain may give $\approx 10^{12}$ and $\approx 10^{18}$, respectively. However, the number of synaptic boutons per dendrite are too inhomogeneous to estimate even in averaged sense, although it is said to be $\approx 10^2 \sim 10^4$. While the number of receptors per synaptic bouton is also difficult to estimate, it is said to be $\approx 10^3 \sim 10^5$ but includes great uncertainty. Therefore, one of the important but quite difficult questions is by what mechanism such numerous degrees of freedom are controlled via neural networks to realize many advanced functions. In particular, the most important point is what and how spatiotemporal concentration of ACh regulates advanced functions of brain via nAChRs, say perceiving, learning, memorizing, attentions, etc.[1] A large number of papers have been published so far, however we are not yet able to have clarified understanding [21].

Now, the following is a summary of problems,

1. The difficulty in accurate evaluations of the causal links between the spatiotemporal concentration of acetylcholine in the brain and a quantitative evaluation of the performance of advanced functioning (e.g., attention, conscious awareness) and mental diseases (e.g., DLB, PD, AD, etc.) [20, 22].
2. The technological problem of measuring the spatial distribution of nAChRs with accurate resolution depending on subunit combinations in nAChRs. [12, 23].
3. The technological problem of measuring the spatiotemporal distribution of acetylcholinesterase (degrading enzyme of ACh) that gives strong influences on local concentration of ACh [24]. Unfortunately, most papers are concerned not to measure the data of acetylcholinesterase but to investigate the effects of acetylcholinesterase inhibitors on diseased patients (AD, LBD, etc.). [25].

## Concluding remarks and issues

Generally speaking, when we have concerns regarding macroscopic behaviors or motions of animals including humans, then experimental observation and theoretical consideration based on contracting the degrees of freedom to ‘small number of macroscopic variables’ enable us to construct phenomenological theory, e.g. non-equilibrium phase transition or information self-organization [26]. In contrast, if neural activity on *mesoscopic scale* are concerned, then neuro-physiological measurements and theoretical consideration are useful for investigating brain functioning [27], although we have to face a difficulty of ‘big data’ obtained by physiological measurements, e.g. EEG, MEG, ECoG, MRI (fMRI, DTI-MR), SPECT, PET, TMS, etc., all of data appear non-stationary (even chaotic) in wide spatiotemporal scale. However, in the both (macroscopic and mesoscopic) approaches, neuromodulators are out of measurements and consideration.

When one wishes to take the effects of neuromodulators into account, there are few options to observe them in humans because ethical principles forbid arbitrary medical treatments. At most, mild effects by pharmacological drugs have been investigated in diseased patients [12]. There are a large number of papers to report global trends of the relation between the concentration of neuromodulators in the brain and behavioral observations among humans [28]. However, a quantitative evaluation of the performance of advanced functioning (e.g., attention, arousal) is very difficult, and moreover, difficult to find certain correspondences between the concentration of neuromodulators in the brain and the pathological symptoms of mental diseased patients.

In contrast, it is possible to make arbitrary designed experiments in animals, e.g. rodents [29], monkeys with use of genetical technology and so on [30] but in these cases, quantitative analysis of behaviors is quite difficult also [31].

Therefore, we have to recognize that many unknown mechanisms still remain hidden behind the deep complexity of brain systems. In these situations surrounded by high hurdle, a candidate to overcome difficulty is ‘functional chaos in systems with large but finite degrees of freedom’, in which there is high degree of redundancy in information processing and/or executing advanced functions from microscopic scale such as biological macro molecules to macroscopic scale, e.g. neural network in the brain. Several examples are shown in the authors’ paper [32–36], where the basic idea may be extended to even mesoscopic and microscopic scale such like the phenomena introduced in the present paper.
